# Association between Cervical Lymph Node Metastasis and the Incidence of Radiation-Induced Hypothyroidism in Nasopharyngeal Carcinoma

**DOI:** 10.1155/2022/5693575

**Published:** 2022-02-01

**Authors:** Ling Zhou, Jia Chen, Chang-Juan Tao, Qing-Feng Zhao, Yu-Ming Chen, Xiao-Zhong Chen, Ming Chen, Zhong-Hua Yu, Yuan-Yuan Chen

**Affiliations:** ^1^Department of Radiation Oncology, Dongguan People's Hospital, Dongguan, China; ^2^Research Center for Healthcare Data Science, Zhejiang Lab, Hangzhou, China; ^3^Department of Radiation Oncology, Cancer Hospital of the University of Chinese Academy of Sciences (Zhejiang Cancer Hospital), Hangzhou, China; ^4^Information Department, Cancer Hospital of the University of Chinese Academy of Sciences (Zhejiang Cancer Hospital), Hangzhou, China; ^5^Department of Radiation Oncology, Sun Yat-Sen University Cancer Center, Guangzhou, China; ^6^Department of Oncology, The Affiliated Hospital of Guangdong Medical University, Zhanjiang, China

## Abstract

**Background:**

It is controversial and unclear how *N*-stage would increase the risk of incidence of hypothyroidism (HT) for patients with nasopharyngeal carcinoma (NPC) after radiotherapy. Our study aimed to explore the correlation between cervical lymph node metastasis and the incidence of HT in NPC.

**Materials and Methods:**

A total of 206 patients with NPC treated at the Cancer Hospital of University of Chinese Academy of Sciences, and their clinical information were retrospectively collected. A series of univariate logistic regression models were performed to explore the association of clinical and lymph node indices with the development of HT. Significant features in univariate analysis were then used to construct three prediction models, for HT prediction using multivariate logistic regression based on Bayesian information criterion. Prediction performance of those models was measured by area under the receiver operating characteristic curve (AUC) using 10-fold cross-validation.

**Results:**

A total of 111 patients developed HT, and the incidence of HT in *N*_2–3_ and *N*_0–1_ patients was 58.82% and 44.29%, respectively. Compared to Model 1 (consisted of pretreatment TSH concentration, thyroid volume, and *N*-stage) whose AUCs were 0.801 and 0.766 in training and validation sets, with *N*-stage be replaced by shortest distance from thyroid, Model 2 achieved more stable AUCs of 0.824 and 0.801. While with numbers of positive lymph nodes in Level IIb additionally added, Model 3 improved its AUCs to 0.841 and 0.813.

**Conclusion:**

The shortest distance between the lymph nodes and thyroid gland and the number of lymph nodes in IIb are better predictors of radiation-induced HT than the *N*-stage.

## 1. Introduction

Nasopharyngeal carcinoma (NPC) is one of the most common malignant tumors in the head and neck region, and it is particularly prevalent in East and Southeast Asian countries [1] Radiotherapy is the main treatment, and cervical lymph nodes are routinely included in the irradiation field because >75% newly treated patients with NPC have cervical lymph node metastasis [2]. The thyroid gland is located in the front of the neck, which makes it an easy target for high-dose radiation exposure, resulting in radiation-induced hypothyroidism (HT) and poor quality of life [3, 4].

The most common symptoms of HT are fatigue, lethargy, cold intolerance, weight gain, constipation, change in voice, and dry skin, although clinical presentation can differ with age and sex. Some patients in the early stage show no obvious symptoms, and hence, treatment is often delayed due to missed diagnosis. However, the cardiovascular, nervous, digestive, and circulatory systems can be involved in the late stage of the disease [5] Therefore, early and timely prediction of HT is important in the prognosis of patients with NPC. At present, the clinical factors including tumor node metastasis (TNM)-stage, pretreatment thyroid-stimulating hormone (TSH) concentration, thyroid volume, sex, and chemotherapy are considered related to HT.

Among the abovementioned factors, TNM-stage is an important basis for treatment and judging prognosis in patients with NPC [6]. Specifically, the *N*-stage represents lymph nodes stage, which refers to the size and extent of cervical lymph node metastasis. In our previous study, *N*-stage was an independent predictor of the incidence of HT [7], which is consistent with other studies [8, 9]. Nonetheless, the relationship between *N*-stage and incidence of HT is still controversial, and as yet, there is no independent study to explore its internal relationship. Some researchers believe that advanced *N*-stage can increase the incidence of HT by influencing the irradiation dose of the neck [8, 9]. However, it is still unknown how the *N*-stage affects the cervical radiation dose, specifically whether the size and number of metastatic lymph nodes or the distance between metastatic lymph nodes and thyroid gland are related to the incidence of HT. Therefore, the purpose of this study was to further explore the internal relationship between the incidence of HT and cervical lymph node metastasis to build the best prediction model for radiation-induced HT and guide individual treatment accordingly.

## 2. Materials and Methods

### 2.1. Patients

In all, 1000 patients with NPC treated at the Cancer Hospital of University of Chinese Academy of Sciences from January 2015 to August 2018 were retrospectively analyzed. Of these, 206 patients who met the inclusion criteria were enrolled. The eligibility criteria included patients with pathologically confirmed primary NPC receiving radical intensity-modulated radiotherapy (IMRT) at our hospital; those with complete clinical data and thyroid function test results before and after radiotherapy; and those with >1 year follow-up with thyroid function tests. The exclusion criteria were patients who had already received radiotherapy before treatment at our hospital or who had a dysfunction with the hypothalamic-pituitary-thyroid (HPT) axis.

### 2.2. Treatment

All patients received radical IMRT. Patients in the supine position were fixed with the head-neck-shoulder thermoplastic mask. The computed tomography simulation (CT-sim) scanned from the skull base to the sternal angle with a thickness of 3 mm. Magnetic resonance imaging (MRI) scan was obtained in the same posture with an immobilization mode (Siemens Verio 3.0T). The CT-sim and MRI scan images were transmitted into RayStation 4.0, and the delineated tumor area on the CT image was combined with the MR image. The gross tumor volume (GTV) included the primary disease (GTVnx) and the metastatic lymph nodes (GTVnd). High-risk clinical target volume (CTV-1) included soft tissue adjacent to GTV. Low-risk CTV (CTV-2) included bilateral lymphatic drainage regions. PGTVnx, PGTVnd, PTV-1, and PTV-2 were established by adding 3 to 5 mm to the GTVnx, GTVnd, CTV-1, and CTV-2, respectively. The prescription dose of PGTVnx, PGTVnd, PTV-1, and PTV-2 was 70.40 Gy, 68.80 Gy, 64.00 Gy, and 54.40 Gy, respectively, and it was completed in 32 fractions. Dose prescription of normal tissue was stated as follows: The maximum dose of the brainstem, optic nerves, and chiasma were 54 Gy, while that of the pituitary was 50 Gy; no dose constraint was given for the thyroid gland during optimization of all IMRT plans. Around 194 patients (94.17%) received platinum-based neoadjuvant chemotherapy, concurrent chemotherapy, or adjuvant chemotherapy, while 12 patients (5.83%) did not receive any chemotherapy.

### 2.3. Redrawing of Metastatic Lymph Nodes

Diagnostic criteria of metastatic lymph nodes: (1) Minimum diameter of lymph nodes with a minimal axial diameter of ≥11 mm in the submandibular and digastric region, the shortest axial diameter of >5 mm in the retropharyngeal lymph nodes, and more than 10 mm in other lymph node regions. (2) Three or more lymph nodes in the same region with a minimal axial diameter of 8 mm in lymph node drainage regions of the tumor. (3) Central necrosis or circular enhancement of lymph nodes. (4) All nodes that show irregular enhancement and mutual fusion should be considered metastatic [10].

According to the diagnostic criteria of lymph node metastasis, a senior doctor redrew the metastatic lymph nodes of 206 patients, divided all lymph nodes according to the new guidelines for delineation of the cervical lymph nodes, and recorded their number and maximum lymph node size (the minimum axial diameter) [11].

### 2.4. Measure the Shortest Distance between Metastatic Lymph Nodes and Thyroid Gland

The thyroid gland was expanded 1, 2, 3, 4, and 5 cm, respectively, and the shortest distance between the metastatic lymph nodes and thyroid gland was recorded, such as 0 to 1 cm, 1 to 2 cm, 2 to 3 cm, 3 to 4 cm, 4 to 5 cm, and >5 cm. If there was no cervical lymph node metastasis, the default measurement was considered as >5 cm.

### 2.5. Thyroid Function Test

HT was defined as TSH concentrations above the reference range (0.380–4.340 IU/mL) and FT4 concentrations within or below the normal range (0.81–1.89 ng/dL) [5]. Thyroid function test was using the electrochemiluminescence method with the SIEMENS ADVIA Centaur XP, followed up at least every 6 months after radiotherapy.

### 2.6. Statistical Analysis

First of all, means and standard deviations were used to describe variables with normal distribution, median (Q25–Q75) were used to describe variables with non-normal distribution, and frequency and percentages were used to describe categorical variables. Their differences were then correspondingly compared using a *t*-test, Wilcoxon rank-sum test (or Mann–Whitney *U* test), and chi-square test (or Fisher's exact probability), respectively. Second, a series of univariate logistic regression models were analyzed to examine which of the clinical and lymph node indices were related to the development of HT. Third, for best prediction of HT and further exploration of its association with cervical lymph nodes, multivariate logistic regression was first constructed based on factors from (1) significant clinical features in the univariate analysis, and then, the *N*-stage in (1) was replaced by significantly positive lymph-node-related factors, respectively (i.e., the shortest distance between the metastatic lymph nodes and thyroid gland together with the size of positive lymph nodes in each neck area, and distance from numbers of metastatic cervical lymph nodes) to build Models 2 and 3. The best fit for those models was determined by statistical fit (Bayesian information criterion [BIC]). In addition, a heat map was drawn to show the collinearity of the number and size of metastatic cervical lymph nodes in each level. To avoid over-fitting, numbers and size of metastatic cervical lymph nodes were separately included in the aforementioned models (i.e., Models 2 and 3) instead of aggregation. Furthermore, model performance was assessed and compared by areas under receiver operating characteristic curve (AUCs) using 10-fold cross-validation. Finally, a nomogram was built for individualized prediction according to the model with best performance. *P* < 0.05 was considered statistically significant. All analyses were performed using *R*, version 3.6.2..

## 3. Results

**Figure 1:**
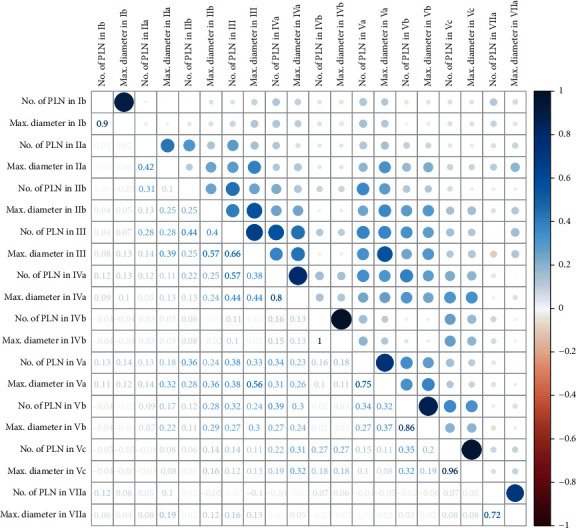
Collinearity of the number and size of metastatic cervical lymph nodes in each level analyzed by heat map. PLN = positive lymph node.

**Figure 2:**
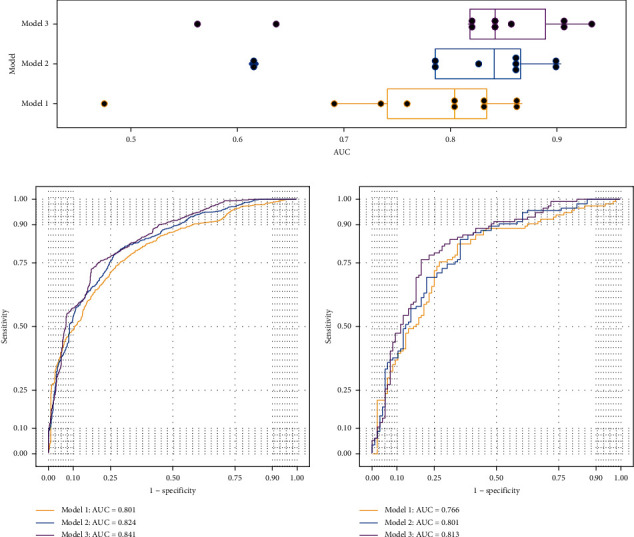
Predictive power comparison between three nomogram models under multivariate analysis: (a) box plot of AUC distribution of the 10 logistic regression models in each 10-fold cross-validation. (b) Training ROC curves for Model 1 (yellow), Model 2 (blue), and Model 3 (purple). The AUCs of the three curves are 0.801, 0.824, and 0.841, respectively. (c) Testing ROC curves for Model 1 (yellow), Model 2 (blue), and Model 3 (purple). The AUCs of the three curves are 0.766, 0.801, and 0.813, respectively.

**Figure 3:**
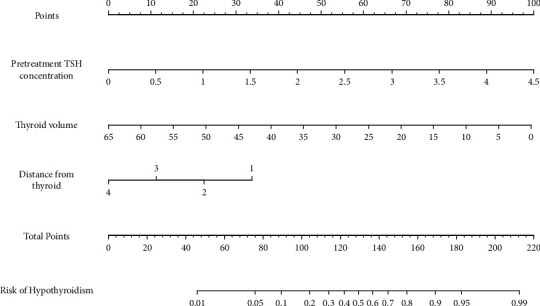
Nomogram-predicted incidence of hypothyroidism based on Model 2. Distance from thyroid = the shortest distance between metastatic lymph nodes and the thyroid gland.

1. Clinical characteristics: The average age of the 206 patients was 51.37 ± 10.71 years. A total of 111 (53.88%) of the 206 patients developed HT due to radiation therapy, and the incidence of HT in *N*_2–3_ patients and *N*_0–1_ patients was 58.82% (80/136) and 44.29% (31/70), respectively, after a median follow-up time of 21 months.  Table 1 shows the general clinical characteristics of 206 patients with NPC.
2. Cervical lymph nodes metastasis: The rate of cervical lymph node metastasis in the group was 96.12% (198/206), and the detailed distribution is as follows: Level IIa was the highest incidence (91.75%) of lymph node metastasis, followed by Level IIb (90.29%). It was found that the number and size of cervical lymph nodes in each level had strong collinearity by heat map, as shown in Figure 1.
3. Univariate analysis for HT. Univariate analysis showed that sex, *N*-stage, pretreatment TSH concentration, thyroid volume, and the shortest distance between metastatic lymph nodes and thyroid gland were correlated with HT. In addition, the number of metastatic lymph nodes in Levels IIa, IIb, III, IVa, and Va and the maximum diameter of lymph nodes in Levels IIb and IVa were also associated with HT. Among them, advanced *N*-stage (*N*_2–3_), high pretreatment TSH concentration, small thyroid volume, large distance between the metastatic lymph nodes and thyroid gland, and large number of metastatic lymph nodes in Level IIb were the risk factors of HT. There was no significant difference in other factors such as age, clinical stage, and chemotherapy (Table 2).
4. Multivariate analysis and prediction model: Based on BIC, pretreatment TSH concentration, thyroid volume, and *N*-stage were built on the best clinical-based model (Model 1 in Table 3) to predict the incidence of radiation-induced HT. Compared to Model 1 whose AUCs were 0.801 and 0.766 in training and validation sets, with *N*-stage be replaced by shortest distance from thyroid, Model 2 achieved more stable AUCs of 0.824 and 0.801. While with numbers of positive lymph nodes in Level IIb additionally added, Model 3 improved its AUCs to 0.841 and 0.813 (Figure 2).
5. Nomogram prediction model: With comprehensive consideration of the prediction performance and simplicity of models, a nomogram based on three-variables was constructed, including the pretreatment TSH concentration, thyroid volume, and the shortest distance between the metastatic lymph nodes and thyroid gland. Model 2 was regarded as the best model of radiation-induced HT prediction, and individualized prediction is shown in Figure 3.

## 4. Discussion

HT is one of the common side effects of NPC after radiotherapy, with an incidence of 20% to 60%, which mainly occurred 1 to 2 years after radiotherapy [12, 13]. Thyroid hormone replacement with levothyroxine is the standard treatment for patients with HT. However, a substantial proportion of patients treated with levothyroxine have persistent complaints [5], which has prompted the question of whether the incidence of HT can be predicted early, in order to screen high-risk patients and improve their quality of life as much as possible. At present, there are few studies on the risk factors related to radiation-induced HT, and its incidence is mainly related to radiation dose, chemotherapy, clinical stage, and other factors [4, 14]. Our previous study found that *N*-stage, radiation dose, and volume of thyroid are independent predictors of HT, combined with *N*-stage, dosimetric parameters, and volume to build an NTCP model [7], but the performance was not very good, which was also the limitation of most similar studies. To our best knowledge, this is the first retrospective study to analyze the number, size, and the shortest distance between the metastatic lymph nodes and thyroid and the correlation between these and the incidence of HT. Furthermore, we also constructed a prediction model of HT based on the related factors of metastatic lymph nodes. The results showed that the shortest distance between the metastatic lymph nodes and thyroid achieved the best prediction of HT and improved the prediction performance. In addition, the NTCP model with the number of lymph nodes in Level IIb also showed good performance.

The correlation between *N*-stage and HT is still controversial. Fujiwara et al. [9] reported that only the radiation field that included the whole neck had a significant correlation with the development of HT, as irradiation of cervical lymph nodes can increase the incidence of HT in patients with head and neck cancer. Huang et al. [15] retrospectively analyzed 345 patients with NPC treated with IMRT and found that *N*_1–3_ patients were more likely to develop HT than *N*_0_ patients, but the internal relationship between lymph node metastasis and the incidence of HT was not explored. In contrast with the aforementioned conclusion, Koc and Capoglu [16] suggested that the clinical stage of NPC has no effect on the incidence of HT. In our previous study, *N*-stage played a major role in the NTCP model for predicting HT in NPC patients, and the incidence of HT in *N*_2–3_ patients and *N*_0–1_ patients was 58.82% and 44.29%, respectively [7]. The results of this study showed that *N*-stage can predict the incidence of HT again, and patients with advanced *N*-stage will inevitably receive higher irradiated volume of the tumor, resulting in a higher risk of thyroid irradiation. Model 1 can predict the incidence of HT by including *N*-stage, thyroid volume, and pretreatment TSH concentration. Compared with Model 1, Model 2 involved the shortest distance between the metastatic lymph nodes and thyroid gland instead of *N*-stage and achieved a better and more stable prediction, with its AUC increasing from 0.801 to 0.824 and 0.766 to 0.801 in the training and test sets, respectively. This probably indicted that the shortest distance between the metastatic lymph nodes and thyroid gland has a greater effect on HT than *N*-stage, even though both related factors may affect the incidence of HT through radiation dose. *N*-stage cannot reflect the minimum distance between lymph nodes and thyroid gland. In addition, compared with Model 2, Model 3 included not only all the factors in Model 2 but also the number of metastatic lymph nodes in Level IIb, which also increased the AUC values of the training and test sets from 0.824 to 0.841 and 0.801 to 0.813, respectively. This suggested that the number of lymph nodes in Level IIb could predict the incidence of HT, which may be related to most of the patients with advanced *N*-stage who had a higher rate of lymph node metastasis in Level IIb. Therefore, when establishing the NTCP model of radiation-induced HT in NPC, it may be necessary to consider the effect of the shortest distance between lymph nodes and thyroid gland and the number of lymph nodes in Level IIb. In a word, considering the prediction effect and simplicity of the model, Model 2 is the best for building an NTCP model for HT in NPC patients.

Consistent with the results of previous studies [13, 17], we found that thyroid volume and pretreatment TSH concentration are independent predictors of HT, as patients with smaller thyroid volume are more likely to develop HT. Moreover, thyroid volume is an independent predictor of HT because a smaller thyroid volume and less thyroid hormone storage naturally lead to higher TSH value [17]. In addition, high TSH concentration before radiotherapy was also a risk factor for HT; the higher the pretreatment TSH concentration, the higher the incidence of HT. In clinical practice, patients with small thyroid volume; high TSH concentration before radiotherapy; and metastatic lymph nodes close to the thyroid gland should be closely followed-up for thyroid function. The nomogram based on Model 2 visualizes the individual prediction, which has clinical guiding significance.

Our study has some limitations. First, the median follow-up (21 months) was relatively short, which may have negatively influenced the evaluation of long-term side effects of radiation-induced HT. Second, there is an inevitable bias because of the retrospective nature of the study. Third, the number of patients in this study was relatively small (*n* = 206). Thus, it is necessary to design a prospective, long-term follow-up study in a larger patient cohort to build a better prediction model of radiation-induced HT.

## 5. Conclusion

The shortest distance between metastatic lymph nodes and the thyroid gland and/or the number of lymph nodes in Level IIb could better predict radiation-induced HT than the *N*-stage.

## Figures and Tables

**Table 1:** The general clinical characteristics of 206 patients with nasopharyngeal carcinoma.

Variables	Euthyroidism	Hypothyroidism	*P*
Sex (%)			0.004^*∗∗*^
Male	73 (76.8)	63 (56.8)	
Female	22 (23.2)	48 (43.2)	
Age (mean [SD])	52.81 (10.61)	50.14 (10.68)	0.075
Pretreatment TSH concentration	1.29 (0.92, 1.74)	2.29 (1.58, 3.06)	<0.001^*∗∗∗*^
Volume (cm^3^)	16.16 (13.43, 19.98)	12.82 (10.79, 16.13)	<0.001^*∗∗∗*^
*T*-stage (%)			1
*T*1–2	18 (18.9)	21 (18.9)	
*T*3–4	77 (81.1)	90 (81.1)	
*N*-stage (%)			0.067
*N*0–1	39 (41.1)	31 (27.9)	
*N*2–3	56 (58.9)	80 (72.1)	
*M*-stage (%)			0.656
*M*0	92 (96.8)	105 (94.6)	
*M*1	3 (3.2)	6 (5.4)	
Clinical stage (%)			0.981
I–II	7 (7.4)	7 (6.3)	
III–IV	88 (92.6)	104 (93.7)	
Neoadjuvant chemotherapy (%)			
No	7 (7.4)	5 (4.5)	0.564
Yes	88 (92.6)	106 (95.5)	
Distance from thyroid (%)			0.005^*∗∗*^
0–1 cm	13 (13.7)	29 (26.1)	
1–2 cm	16 (16.8)	30 (27.0)	
2–3 cm	31 (32.6)	31 (27.9)	
>3 cm	35 (36.8)	21 (18.9)	
Number. Level Ib (%)			0.538
0	87 (91.6)	100 (90.1)	
1-2	7 (7.4)	11 (9.9)	
≥3	1 (1.1)	0 (0.0)	
Number. Level IIa (%)			0.046^*∗*^
0	12 (12.6)	5 (4.5)	
1–2	69 (72.6)	95 (85.6)	
≥3	14 (14.7)	11 (9.9)	
Number. Level IIb (%)			0.001^*∗∗*^
0	16 (16.8)	4 (3.6)	
1–2	48 (50.5)	78 (70.3)	
≥3	31 (32.6)	29 (26.1)	
Number. Level III (%)			0.048^*∗*^
0	36 (37.9)	27 (24.3)	
1–2	42 (44.2)	51 (45.9)	
≥3	17 (17.9)	33 (29.7)	
Number. Level IVa (%)			0.006^*∗∗*^
0	70 (73.7)	58 (52.3)	
1–2	21 (22.1)	41 (36.9)	
≥3	4 (4.2)	12 (10.8)	
Number. Level IVb (%)			0.173
0	95 (100.0)	107 (96.4)	
1–2	0 (0.0)	4 (3.6)	
Number. Level Va (%)			0.04^*∗*^
0	64 (67.4)	58 (52.3)	
1–2	30 (31.6)	47 (42.3)	
≥3	1 (1.1)	6 (5.4)	
Number. Level Vb (%)			0.163
0	81 (85.3)	85 (76.6)	
1–2	14 (14.7)	26 (23.4)	
Number. Level Vc (%)			1
0	93 (97.9)	108 (97.3)	
1–2	2 (2.1)	3 (2.7)	
Number. Level VIIa (%)			0.898
0	24 (25.3)	30 (27.0)	
1–2	71 (74.7)	81 (73.0)	
Maxdiameter. Level Ib (%)			0.48
0 cm	87 (91.6)	100 (90.1)	
0–1 cm	7 (7.4)	7 (6.3)	
≥1 cm	1 (1.1)	4 (3.6)	
Maxdiameter. Level IIa (%)			0.073
0 cm	12 (12.6)	5 (4.5)	
0–1 cm	56 (58.9)	65 (58.6)	
≥1 cm	27 (28.4)	41 (36.9)	
Maxdiameter. Level IIb (%)			0.006^*∗∗*^
0 cm	16 (16.8)	4 (3.6)	
0–1 cm	41 (43.2)	57 (51.4)	
≥1 cm	38 (40.0)	50 (45.0)	
Maxdiameter. Level III (%)			0.07
0 cm	36 (37.9)	27 (24.3)	
0–1 cm	47 (49.5)	72 (64.9)	
≥1 cm	12 (12.6)	12 (10.8)	
Maxdiameter. Level IVa (%)			0.007^*∗∗*^
0 cm	70 (73.7)	58 (52.3)	
0–1 cm	22 (23.2)	46 (41.4)	
≥1 cm	3 (3.2)	7 (6.3)	
Maxdiameter. Level IVb (%)			0.251
0 cm	95 (100.0)	107 (96.4)	
0–1 cm	0 (0.0)	2 (1.8)	
≥1 cm	0 (0.0)	2 (1.8)	
Maxdiameter. Level Va (%)			0.079
0 cm	64 (67.4)	58 (52.3)	
0–1 cm	25 (26.3)	45 (40.5)	
≥1 cm	6 (6.3)	8 (7.2)	
Maxdiameter. Level Vb (%)			0.238
0 cm	81 (85.3)	85 (76.6)	
0–1 cm	12 (12.6)	20 (18.0)	
≥1 cm	2 (2.1)	6 (5.4)	
Maxdiameter. Level Vc (%)			1
0 cm	93 (97.9)	108 (97.3)	
0–1 cm	2 (2.1)	2 (1.8)	
≥1 cm	0 (0.0)	1 (0.9)	
Maxdiameter. Level VIIa (%)			
0 cm	24 (25.3)	30 (27.0)	0.653
0–1 cm	48 (50.5)	60 (54.1)	
≥1 cm	23 (24.2)	21 (18.9)	
Number. all	7.00 (5.00, 10.00)	9.00 (6.00, 11.00)	0.008^*∗∗*^
Maxdiameter. all	1.04 (0.81, 1.38)	1.15 (0.94, 1.54)	0.039^*∗*^

The values in the table are *N* (%) or median (Q25–Q75) unless otherwise indicated; Maxdiameter: maximum diameter; ^*∗*^*P* < 0.05, ^*∗∗*^*P* < 0.01, ^*∗∗∗*^*P* < 0.001.

**Table 2:** Univariate analysis of radiation-induced HT.

Variables		Beta		OR (95% CI)		*P*	
Age		−0.02		0.98 (0.95–1.00)		0.076	
Sex		0.93		2.53 (1.38–4.64)		0.003^*∗∗*^	
Pretreatment TSH concentration		1.2		3.33 (2.24–4.95)		<0.001^*∗∗∗*^	
Volume (cm^3^)		−0.13		0.88 (0.83–0.94)		<0.001^*∗∗∗*^	
T-Stage (T1–2 vs. T3–4)		0.00		1.00 (0.50–2.01)		0.996	
N-stage (N2–3 vs. N0–1)		0.59		1.80 (1.00–3.22)		0.048^*∗*^	
M-Stage (M1 vs. M0)		0.56		1.75 (0.43–7.21)		0.437	
Clinical stage (III–IV vs I–II)		0.17		1.18 (0.40–3.50)		0.763	
Neoadjuvant chemotherapy (yes vs. no)		0.52		1.69 (0.52–5.50)		0.386	
Distance from thyroid 1–2 cm (vs. 0–1 cm)		−0.17		−0.17 (0.34–2.05)		0.703	
Distance from thyroid 2–3 cm (vs. 0–1 cm)		−0.8		−0.80 (0.20–1.02)		0.056	
Distance from thyroid >3 cm (vs. 0–1 cm)		−1.31		−1.31 (0.12–0.63)		0.002 ^*∗∗*^	
N.Ib (1∼2 vs 0)		0.31		0.31 (0.51–3.68)		0.536	
N.Ib (3+ vs 0)		−14.71		−14.71 (0.00-Inf)		0.987	
N.IIa (1∼2 vs 0)		1.2		1.20 (1.11–9.81)		0.031^*∗*^	
N.IIa (3+ vs 0)		0.63		0.63 (0.51–6.98)		0.342	
N.IIb (1∼2 vs 0)		1.87		1.87 (2.05–20.59)		0.001^*∗∗*^	
N.IIb (3+ vs 0)		1.32		1.32 (1.12–12.51)		0.032^*∗*^	
N.III (1∼2 vs 0)		0.48		0.48 (0.85–3.09)		0.143	
N.III (3+ vs 0)		0.95		0.95 (1.20–5.58)		0.015^*∗*^	
N.IVa (1∼2 vs 0)		0.86		0.86 (1.25–4.43)		0.008^*∗∗*^	
N.IVa (3+ vs 0)		1.29		1.29 (1.11–11.83)		0.033^*∗*^	
N.IVb (1∼2 vs 0)		15.45		5112076.84 (0.00-Inf)		0.983	
N.Va (1∼2 vs 0)		0.55		0.55 (0.97–3.09)		0.064	
N.Va (3+ vs 0)		1.89		1.89 (0.77–56.65)		0.084	
N.Vb (1∼2 vs 0)		0.57		1.77 (0.86–3.63)		0.119	
N.Vc (1∼2 vs 0)		0.26		1.29 (0.21–7.90)		0.782	
N.VIIa (1∼2 vs 0)		−0.09		0.91 (0.49–1.70)		0.774	
Maxdiameter.Ib (0, 1) cm		−0.14		−0.14 (0.29–2.58)		0.802	
Maxdiameter.Ib ≥ 1 cm		1.25		1.25 (0.38–31.69)		0.269	
Maxdiameter.IIa (0, 1) cm		1.02		1.02 (0.92–8.39)		0.069	
Maxdiameter.IIa ≥ 1 cm		1.29		1.29 (1.15–11.52)		0.028^*∗*^	
Maxdiameter.IIb (0, 1) cm		1.72		1.72 (1.73–17.86)		0.004^*∗∗*^	
Maxdiameter.IIb ≥ 1 cm		1.66		1.66 (1.63–17.03)		0.006^*∗∗*^	
Maxdiameter.III (0, 1) cm		0.71		0.71 (1.10–3.80)		0.024^*∗*^	
Maxdiameter.III ≥ 1 cm		0.29		0.29 (0.52–3.42)		0.55	
Maxdiameter.IVa (0, 1) cm		0.93		0.93 (1.36–4.67)		0.003^*∗∗*^	
Maxdiameter.IVa ≥ 1 cm		1.04		1.04 (0.70–11.38)		0.146	
Maxdiameter.IVb (0, 1) cm		15.45		15.45 (0.00-Inf)		0.988	
Maxdiameter.IVb ≥ 1 cm		15.45		15.45 (0.00-Inf)		0.988	
Maxdiameter.Va (0, 1) cm		0.69		0.69 (1.09–3.64)		0.026^*∗*^	
Maxdiameter.Va ≥ 1 cm		0.39		0.39 (0.48–4.49)		0.498	
Maxdiameter.Vb (0, 1) cm		0.46		0.46 (0.73–3.46)		0.244	
Maxdiameter.Vb ≥ 1 cm		1.05		1.05 (0.56–14.58)		0.206	
Maxdiameter.Vc (0, 1) cm		−0.15		−0.15 (0.12–6.23)		0.882	
Maxdiameter.Vc ≥ 1 cm		14.42		14.42 (0.00-Inf)		0.987	
Maxdiameter.VIIa (0, 1) cm		0.00		−0.00 (0.52–1.93)		1	
Maxdiameter.VIIa ≥ 1 cm		−0.31		−0.31 (0.33–1.62)		0.441	
*N*. all		0.1		1.11 (1.03–1.19)		0.008^*∗∗*^	
Maxdiameter.all		0.59		1.81 (1.03–3.18)		0.04^*∗*^	

OR = odds ratio; CI = confidence interval; Distance from thyroid = the shortest distance between metastatic lymph nodes and the thyroid gland; *N* = number. ^*∗*^*P* < 0.05, ^*∗∗*^*P* < 0.01, ^*∗∗∗*^*P* < 0.001.

**Table 3:** Multivariate analysis of radiation-induced HT.

Variables (ref)	Model 1			Model 2			Model 3		
	Beta	OR (95% CI)	*P*	Beta	OR (95% CI)	*P*	Beta	OR (95% CI)	*P*
Constant	−1.08	0.34 (0.08–1.46)	0.146	0.83	2.30 (0.48–11.12)	0.300	−0.67	0.51 (0.05–4.89)	0.563
*N* stage	0.79	2.20 (1.12–4.33)	0.023						
Pretreatment TSH	1.14	3.12 (2.07–4.72)	<0.001	1.23	3.41 (2.20–5.29)	<0.001	1.26	3.54 (2.25–5.57)	<0.001
Thyroid volume	−0.09	0.91 (0.85–0.98)	0.010	−0.08	0.92 (0.86–0.99)	0.018	−0.09	0.92 (0.85–0.99)	0.020
Shortest distance from thyroid				−0.62	0.54 (0.39–0.74)	<0.001	−0.56	0.57 (0.41–0.80)	0.001
Numbers of positive lymph nodes in IIb area									
1–2 (ref: 0)							1.69	5.42 (1.40–20.97)	0.014
3+ (ref: 0)							0.79	2.19 (0.52–9.19)	0.282

## Data Availability

The data sets supporting the conclusions of this article are included within the article.
